# Towels as They Come from the Laundry

**Published:** 1904-06

**Authors:** P. E. Clarkson


### TOWELS AS THEY COME FROM THE LAUNDRY.

By P. E. Clarkson.

First, we decide to try if the ordinary linen, as we get it from the laundry, is really aseptic. Accordingly, with a pair of scissors and a pair of pliers, both absolutely sterile, we snipped a piece out of the inner folds of a towel, and lodged it with every precaution on top of some sterile glucose agar, pressing the particle gently against the surface, and replaced the sterile cotton plug in the test tube, everything having been previously sterilized except the bit of linen. Then, with the same care, we cut off a bit of the fringe of the same towel (this towel had been lying in the locker a week or more, but never used). Of this we made a stab in some agar medium, pressing it with a sterile needle down to the bottom of the test tube, so that it would be away from oxygen. The two tubes were placed in the incubator and watched daily, but even at the end of the seventh day there was no sign of any growth whatever. This, we are pleased to think, goes to prove that we can depend upon our laundry really being sterile, as germs of any kind had every chance to develop if they were present.—*Dominion.*

